# Interaction between cognitive leisure activity and long-chain polyunsaturated fatty acid intake on global cognitive decline in a Japanese longitudinal cohort study: National Institute for Longevity Sciences-Longitudinal Study of Aging

**DOI:** 10.1186/s12877-021-02359-8

**Published:** 2021-07-27

**Authors:** Chika Horikawa, Rei Otsuka, Yukiko Nishita, Chikako Tange, Yuki Kato, Takao Tanaka, Tomohiro Rogi, Hiroshi Shibata, Fujiko Ando, Hiroshi Shimokata

**Affiliations:** 1 Department of Epidemiology of Aging, National Center for Geriatrics and Gerontology, 7-430 Morioka-cho, 474-8511 Obu-City, Aichi Japan; 2 Institute for Health Care Science, Suntory Wellness Limited, 8-1-1 Seikadai, Seika-cho, Soraku- gun, 619-0284 Kyoto, Japan; 3 Faculty of Health and Medical Sciences, Aichi Shukutoku University, 2-9 Katahira, 480-1197 Nagakute- city, Aichi Japan; 4 Graduate School of Nutritional Sciences, Nagoya University of Arts and Sciences, 57 Takenoyama, Iwasaki-cho, 470-0196 Nisshin-city, Aichi Japan

**Keywords:** long-chain polyunsaturated fatty acids, cognitive leisure activities, NILS-LSA

## Abstract

**Background:**

There is a growing interest in the significance of adopting a variety of lifestyle habits for maintaining cognitive function among older adults. A lifestyle that is easy to modify, simple, and less burdensome for older people is ideal. We investigated the longitudinal association between global cognitive decline and cognitive leisure activities (CLAs) combined with long-chain polyunsaturated fatty acids (LCPUFAs) intake.

**Methods:**

The National Institute for Longevity Sciences-Longitudinal Study of Aging (NILS-LSA) enrolled community-dwelling middle-aged and older men and women who were randomly selected from Obu-City and Higashiura Town, Aichi, Japan. Baseline data (2006–2008), including CLAs and dietary intake, were obtained from 517 participants (aged 60–84 years) with normal cognition. Global cognitive decline, defined as the Mini-Mental State Examination (MMSE) score ≤ 27, was assessed at baseline and four years later. Interaction between CLAs and LCPUFAs on cognitive decline was investigated using a multiple logistic analysis with adjustment for confounders. CLA engagement and LCPUFA intake were divided into high and low groups according to the frequency at which each participant engaged in the activity and the median intake level according to sex, respectively.

**Results:**

A significant interaction was detected for the combination of CLA engagement and LCPUFA intake. Logistic regression coefficients revealed significant interactions when participants engaged in more than five CLA varieties. One of the CLAs, art appreciation, produced a significant main effect against cognitive decline and a significant interaction in combination with LCPUFA intake. The major LCPUFAs—docosahexaenoic acid and arachidonic acid—also exhibited a significant interaction. The combination of high LCPUFA intake and high art appreciation frequency yielded a lower adjusted odds ratio for cognitive decline than the combination of low LCPUFA and low art appreciation [0.25 (95 % confidence intervals, 0.11–0.56)].

**Conclusions:**

Preserving cognitive function might be associated with a combination of varied and high-frequency engagement in CLAs combined with high LCPUFA intake.

## Background

Dementia is a serious public health problem worldwide. The latest World Health Organization guidelines recommend adopting healthy lifestyle behaviors, such as physical activity, smoking cessation, and a balanced diet, for preserving cognition [1]. Recent clinical trials investigating interventions for cognitive preservation have focused on the value of addressing lifestyle practices with multidomain interventions [2–4]. However, the choice of specific lifestyle factors to combine remains unclear due to the inconsistency in the results across studies and the availability of interventions.

Older people have more time to engage in leisure activities compared with younger individuals. Physical activity is often recommended as a preventive measure for preventing cognitive decline [1]. However, cognitive leisure activities (CLAs) with lower physical burdens may be more suitable for older adults. A systematic review defined CLAs as activities for enjoyment or well-being that cause intellectual stimulation and suggested the improvement of cognitive function through CLA interventions [5]. The CLAs assessed in the study included arts, writing, and board games. A meta-analysis of observational studies suggested that CLAs lowered the dementia risk not only among people with good cognitive health, but also among those with mild cognitive impairment (MCI) [6, 7].

Diet is another modifiable lifestyle factor. Long-chain polyunsaturated fatty acids (LCPUFAs), such as docosahexaenoic acid (DHA), eicosapentaenoic acid (EPA), and arachidonic acid (ARA), are important nutrients present in marine and animal food products that contribute to maintaining health. In addition, LCPUFAs are major components of the brain. Despite the importance of brain LCPUFAs, their levels have been reported to decrease with age [8–10]. In animal studies, LCPUFAs were shown to be involved in the regulation and maintenance of brain function, such as neural membrane fluidity, synaptic plasticity, and neurogenesis [11–17]. Moreover, some intervention studies have indicated that supplemental LCPUFAs improve cognitive function in older adults [18–20].

The Finger Study is a recent and well-known clinical trial that evaluated cognitive function maintenance using a multidomain intervention, including fish consumption (rich in LCPUFAs) and cognitive training as interventions [2]. A combination of activation and nutrients could be crucial in maintaining cognitive function. As LCPUFAs levels in the brain decrease with age and play an important role in cognition, we hypothesized that LCPUFA supplementation combined with engagement in activities that stimulate cognitive function is more effective in maintaining cognitive function in older adults than practicing either alone. In other words, the combination of two beneficial lifestyle factors may produce an additive or synergistic effect on maintaining cognitive function in older people.

However, no epidemiological studies have addressed the impact of the combination of CLAs with LCPUFAs on cognitive decline. In this longitudinal study, we focused on the association between CLA combined with LCPUFA intake and cognitive decline in community-dwelling Japanese older adults.

## Methods

### Study design

The data analyzed in the present longitudinal study were obtained from the National Institute for Longevity Sciences-Longitudinal Study of Aging (NILS-LSA), a Japanese population-based, prospective cohort study. Participants in the first wave (1997–2000) of the NILS-LSA included ~ 2,300 men and women aged 40–79 years [21]. Participants were followed up every 2 years until the seventh wave of the examination, which was completed in 2012, and a postal survey is ongoing. Participants included randomly selected, age- and sex-stratified individuals from Obu-City and Higashiura Town in Aichi Prefecture. When participants could not attend the follow-up investigations, the same number of age- and sex-matched random samples were recruited, excluding individuals aged > 79 years.

### Study population

**Fig. 1:**
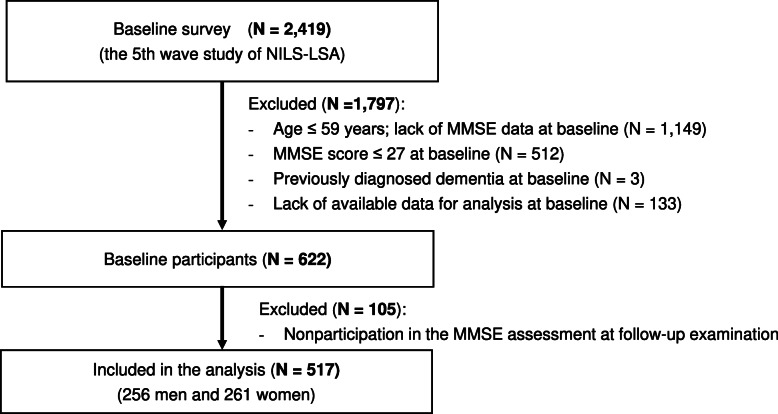
Participants included in this longitudinal study. MMSE, Mini-Mental State Examination; NILS-LSA, National Institute for Longevity Sciences-Longitudinal Study of Aging; N, number

Baseline data were collected from the fifth wave survey (2006–2008), which included 2,419 men and women aged 40 years or older. The follow-up period was 4 years, representing the period from the fifth to the seventh wave (2010–2012). We excluded the following individuals: age ≤ 59 years at baseline because the Mini-Mental State Examination (MMSE) is assessed only in older participants (age ≥ 60 years); those who failed to participate in MMSE assessment at the fifth and seventh wave survey; those with MMSE scores ≤ 27 at baseline; those with a self-reported history of previously diagnosed dementia at baseline; and those with missing data at baseline for adjusted variables in the statistical analysis. By excluding those who failed to meet the inclusion criteria or participate in the follow-up MMSE assessment, 517 participants were included in this analysis (Fig. 1). The Committee of Ethics of Human Research of the National Center for Geriatrics and Gerontology approved this study (approval number:1322), and all methods were performed in accordance with relevant guidelines and regulations. Written informed consent was obtained from all participants.

### Cognitive assessment

Cognitive function was assessed using the Japanese version of the MMSE [22, 23]. The MMSE, which is a brief measure of global cognition and a screening test for dementia, is the most commonly administered test in clinical and research settings. Trained psychologists and researchers administered the test at each wave. The MMSE score ranges from 0 to 30, with higher scores indicating better cognitive function. The participants in the NILS-LSA (age ≥ 60 years) were relatively highly educated [24], and it has been determined that the standard MMSE cut-off score of 23/24 would not provide an adequate classification of highly educated older individuals [24–26]; a higher cut-off score of 26/27 or 27/28 has been recommended for highly educated individuals [25]. Therefore, we defined cognitive decline as an MMSE score of ≤ 27.

### Cognitive leisure activities

The items of the questionnaire for leisure activities in the NILS-LSA were developed by referring to various administrative surveys and a study [27] that surveyed the leisure activities of older adults. CLAs vary according to country and culture. A Japanese group, the Japanese longitudinal cohort study of Septuagenarians, Octogenarians, and Nonagenarians Investigation with Centenarians, was comprehensively surveyed and various Japanese leisure activities were categorized [28]. Reportedly, a categorized group, named “hobby activities” was associated with decreased MCI risk [28]. The “hobby activities” part of the questionnaire used in the above Japanese cohort includes six leisure activities, which correspond to the questionnaire used in NILS-LSA. The six CLAs were reading newspapers, reading books, writing (e.g., diary writing as a hobby), creative activities (e.g., calligraphy, painting, photography, and Japanese dressmaking), art appreciation (e.g., theater visits, watching movies, and music appreciation), and gardening (e.g., home gardening and Japanese bonsai gardening). A research psychometrist assessed the frequency with which each participant engaged in each CLA using a questionnaire survey with the following responses: “never,” “once or several times a year,” “once or several times a month,” “once a week,” “several times a week,” or “every day.” Participants were asked to provide information about their engagement in these activities during the year before study participation.

### Nutritional assessment

Nutritional intake per day, including mean energy intake and fatty acid intake, was assessed using a 3-day dietary record after participation. The dietary record was completed over three continuous days (2 weekdays and 1 day of the weekend) [29]. Most participants completed the records at home and returned them within one month. Either the food was weighed separately on a kitchen scale before being cooked or portion sizes were estimated. Participants took photographs of their meals before and after eating. The average 3-day food and nutrient intake was calculated according to the Standard Tables of Food Composition in Japan, 2010 [30]. Alcohol intake in the previous year was assessed using a food-frequency questionnaire., We focused on three fatty acids, DHA, EPA, and ARA, as representative LCPUFAs because of their relatively high intake.

### Other measurements

The questionnaires were collected on the examination day. Both weight and height were measured on the examination day, to calculate body mass index (BMI). Trained interviewers used a questionnaire for assessing physical activity regarding the intensity and frequency of activity over the preceding year [31]. The mean amount of total physical activity per day was calculated (metabolic equivalents [MET], MET × min/day). The depressive tendency was assessed using the Japanese version of the CES-D questionnaire [32, 33]. Participants completed the CES-D questionnaire at home to assess their level of depression in the previous week and brought it with them on the examination day. A self-completed questionnaire, which was provided approximately 2 weeks before the examination day, was used to collect information on the participant’s history of hypertension, hyperlipidemia, ischemic heart disease, stroke, and diabetes (yes/no); education (≤ 9, 10–12, or ≥ 13 years of school); employment status (no occupation or household labor, irregular employment, and regular employment); marital status (yes/no); and smoking status (yes/no).

### Statistical analyses

All analyses were conducted from 2019 to 2020 using the Statistical Analysis System version 9.3 software (SAS Institute, Cary, NC, USA). The confounding variables were sex; age; MMSE score; BMI; education level; marital status; smoking status; alcohol consumption; energy intake; physical activity; employment status; depressive tendency (assessed using the CES-D score); and history of hypertension, hyperlipidemia, ischemic heart disease, stroke, and diabetes at the baseline. Differences in characteristics, frequency of CLA, and fatty acid intake among participants with and without cognitive decline were assessed using the chi-squared test (categorical variables) or Student’s *t*-test (continuous variables). Fisher’s exact test was used as an alternative to the chi-squared test if the expected counts obtained were < 5. For the analysis of interactions, we divided CLAs into two groups according to the frequency of participation; “at least once or more per month” (high); “once or several times per year or less” (low) [34]. We established high and low LCPUFA groups based on the median LCPUFA intake according to sex. First, the association between cognitive decline and each of the two exposures, CLAs and LCPUFA intake, was estimated using the main effect model. In addition, the total number of CLAs that the individual engaged with high frequency (high) was calculated. Apart from detection of the interactions between each CLA and the intake of each fatty acid, we also investigated the interactions between LCPUFA intake and the total number of CLAs in which the individuals engaged with high frequency. Furthermore, we performed a multiple logistic regression analysis and estimated the odds ratios (ORs) and 95 % confidence intervals (CIs) for the three combinations compared with lower frequencies and intakes. In model I, we adjusted for age and sex. Model II was further adjusted for the above-mentioned confounding variables. Two-sided *P* values < 0.05 were regarded as statistically significant.

## Results

### Baseline characteristics

The mean age was 68.6 (SD, 6.2) years, and 141 (27.3 %) participants showed a cognitive decline after 4 years. Without adjusting for confounders, participants who developed cognitive decline were significantly older, had lesser regular employment, and had lower MMSE scores at baseline (Table 1). More than half of the total participants frequently engaged in CLAs, such as reading the newspaper, reading books, writing, and gardening. The percentage of participation in creative activities and art appreciation was lower than in the remaining four CLAs. Engagement in art appreciation was significantly different between those with and without cognitive decline. Participants with cognitive decline also had a significantly lower intake of saturated fatty acids and MUFAs than those without cognitive decline. The median values of LCPUFA intake that segregated the two groups (high and low) were 1,412 mg/day (men) and 1,071 mg/day (women).

**Table 1:** Baseline characteristics of the participants according to the presence or absence of 4-year period of cognitive decline

Characteristics				Cognitive decline						
	Total			presence			absence			
	Mean		SD	Mean		SD	Mean		SD	*p*-Value
No. of participants (n)	517			141			376			
Sex (n) men	256	(49.5%)		79	(56.0%)		177	(47.1%)		0.07
Age (years)	68.6		6.2	70.0		6.8	68.0		5.8	<0.01
MMSE	28.9		0.8	28.6		0.7	29.1		0.8	<0.01
BMI (kg/m^2^)	22.9		2.8	23.2		2.9	22.7		2.8	0.08
Educational level (n)										0.09
9 years	121	(23.4%)		55	(39.0%)		66	(17.6%)		
10–12 years	228	(44.1%)		48	(34.0%)		180	(47.9%)		
≥13 years	168	(32.5%)		38	(27.0%)		130	(34.6%)		
Married (n)	433	(83.8%)		115	(81.6%)		318	(84.6%)		0.41
Current smoker (n)	41	(7.9%)		9	(6.4%)		32	(8.5%)		0.43
Alcohol consumption^a^ (mL/day)	8.3		14.4	9.9		16.8	7.6		13.3	0.15
Energy intake (kcal/day)	2009		373	2010		391	2009		367	0.97
Total physical activity (METs*min/1000/day)	1.9		0.2	1.9		0.2	1.9		0.2	0.85
Employment (n)										0.03
Unemployed or household labor	261		(50.5%)	81		(57.5%)	180		(47.9%)	
Nonregular employment	87		(16.8%)	24		(17.0%)	63		(16.8%)	
Regular employment	169		(32.7%)	36		(25.5%)	133		(35.4%)	
CES-D	6.6		6.6	6.8		6.8	6.6		6.6	0.76
Medical history (n)^b^										
Stroke	26	(5.0%)		8	(5.7%)		18	(4.8%)		0.68
ypertension	210	(40.6%)		61	(43.3%)		149	(39.6%)		0.45
Ischemic heart disease	31	(6.0%)		10	(7.1%)		21	(5.6%)		0.52
Hyperlipidemia	137	(26.5%)		36	(25.5%)		101	(26.9%)		0.76
Diabetes mellitus	53	(10.3%)		15	(10.6%)		38	(10.1%)		0.86
CLA High frequency (n)^c^										
Reading the newspaper	510	(98.6%)		138	(97.9%)		372	(98.9%)		0.40
Reading books	433	(83.8%)		121	(85.8%)		312	(83.0%)		0.44
Writing	263	(50.9%)		70	(49.6%)		193	(51.3%)		0.73
Creative activities	198	(38.3%)		57	(40.4%)		141	(37.5%)		0.54
Art appreciation	163	(31.5%)		33	(23.4%)		130	(34.6%)		0.02
Gardening	385	(74.5%)		108	(76.6%)		277	(73.8%)		0.50
SFA (g/day)	14.8	4.8		14.4	4.4		15.0	4.9		0.04
MUFA (g/day)	18.2	5.7		17.2	5.5		18.6	5.7		0.02
LCPUFA (g/day)	1.4	0.8		1.4	0.8		1.4	0.8		0.93

Note: Continuous variables, Student’s *t*-test; categorical variables, χ2 test

^a^Alcohol consumption was converted into ethanol content.

^b^Past and present illness

^c^CLA was divided into two groups based on the frequency of participation; “at least once or more per month” (high) “once or several times per year or less” (low)

ARA, arachidonic acid; BMI, body mass index; CES-D, Center for Epidemiologic Studies Depression Scale; DHA, docosahexaenoic acid; EPA, eicosapentaenoic acid; MMSE, Mini-Mental State Examination. MUFA, monounsaturated fatty acid; SFA, saturated fatty acid

### Interaction between cognitive leisure activities and intake of long-chain polyunsaturated fatty acids according to the total number of cognitive leisure activities

Table 2 shows the interaction between the level (high and low) of LCPUFA intake and total variety of CLAs with a high frequency of participation (at least once or more per month). All participants participated with a high frequency in at least one or more out of the six CLAs. No significant interaction was found between the number of CLAs for those who participated in ≥ 1 to ≥ 4 CLAs and LCPUFA intake. The logistic regression coefficients for the interactions between the total variety of CLAs and the level of LCPUFA intake were significant at − 1.003 (SE, 0.507) when performing ≥ 5 activities (*P* = 0.048).

**Table 2:** Interaction between CLA and LCPUFA intake according to the total number of CLAs

Total number of CLAs with high frequency (more than x)	High frequency		Estimate	SE	Interaction *P*-value
(x)	(n)	(%)			
≥1	517	100.0	-	-	-
≥2	500	96.7	0.32	1.20	0.79
≥3	428	82.8	0.34	0.63	0.59
≥4	302	58.4	−0.23	0.46	0.62
≥5	156	30.2	−1.00	0.51	0.05
6	52	10.1	−1.08	0.94	0.25

Note: Multiple logistic analysis adjusted for sex, age, MMSE score, BMI, education level, marital status, smoking status, alcohol consumption, energy intake, physical activity, employment status, depressive tendency, and history of hypertension, hyperlipidemia, ischemic heart disease, stroke, and diabetes

Fatty acid intake was divided into two groups based on the median according to sex. CLA was divided into two groups based on the frequency of participation: “participation of at least once or more per month” (high) and “once or several times per year or less” (low). CLA, cognitive leisure activity; LCPUFA, long-chain polyunsaturated fatty acid

### Main effect of each cognitive leisure activity and intake of long-chain polyunsaturated fatty acids on cognitive decline

One of the CLAs, art appreciation, showed a statistically significant main effect after adjusting for other potential confounders; however, none of the other CLAs exhibited a significant main effect (Table 3). Another exposure, LCPUFA intake, also showed a significant main effect after adjusting for other potential confounders [0.62 (95 % CI, 0.40–0.98)]. DHA, EPA, and ARA did not produce significant main effects [DHA: 0.75 (95 % CI, 0.48–1.18), EPA: 0.73 (95 % CI, 0.47–1.14), ARA: 0.69 (95 % CI, 0.44–1.10)]. In addition, a significant main effect on cognitive decline was detected when the total number of CLAs engaged in by the participant was six [0.43 (95 % CI, 0.19–0.97)].

**Table 3:** Main effect of each CLA on cognitive decline

	High frequency of CLA^a^				
CLAs	(n)	(%)	OR^b^	95% CI	
Reading the newspaper	510	98.6	0.657	0.235	1.838
Reading books	433	83.8	1.468	0.776	2.770
Writing	263	50.9	0.803	0.514	1.255
Creative activities	198	38.3	1.140	0.723	1.799
Art appreciation	163	31.5	0.556	0.333	0.929
Gardening	385	74.5	1.284	0.815	2.024

Note: Multiple logistic analysis adjusted for sex, age, MMSE score, BMI, education level, marital status, smoking status, alcohol consumption, energy intake, physical activity, employment status, depressive tendency, and history of hypertension, hyperlipidemia, ischemic heart disease, stroke, and diabetes

^a^CLAs were divided into two groups based on the frequency of participation; “at least once or more per month” (high); “once or several times per year or less” (low)

^b^OR for cognitive decline compared with low-frequency CLAs as a reference

CI, confidence interval; CLA, cognitive leisure activity; MMSE, Mini-Mental State Examination

### Interaction between the intake of each long-chain polyunsaturated fatty acid and each cognitive leisure activity on cognitive decline

Interaction of art appreciation with each LCPUFA, except EPA, was also observed (Table 4). Reading the newspaper was excluded from the table because it was in a quasi-complete separation state in the analysis. Among the five activities, art appreciation showed a significant interaction with LCPUFA, DHA, and ARA intake. Furthermore, the interaction between art appreciation and LCPUFA was significant at various delimitations of frequency, not only “once or several times a month or more” but also “once a week” (p = 0.0131) and “several times a week” (p = 0.0341).

**Table 4:** Interaction between each CLA and intake of each fatty acid

CLA	LCPUFA			DHA			EPA			ARA		
	Estimate	SE	Interaction	Estimate	SE	Interaction	Estimate	SE	Interaction	Estimate	SE	Interaction
			*P*-Value			*P*-Value			*P*-Value			*P*-Value
Reading books	0.66	0.69	0.34	0.81	0.69	0.24	0.69	0.69	0.32	−0.30	0.65	0.65
Writing	−0.35	0.46	0.45	−0.13	0.45	0.78	−0.25	0.46	0.58	0.05	0.45	0.91
Creative activities	−0.36	0.46	0.43	−0.38	0.46	0.40	−0.38	0.46	0.41	−0.41	0.46	0.37
Art appreciation	−1.15	0.53	0.03	−1.22	0.53	0.02	−0.76	0.51	0.14	−1.29	0.54	0.02
Gardening	0.22	0.52	0.67	0.52	0.52	0.32	0.04	0.512	0.94	−0.54	0.52	0.29

Note: Multiple logistic analysis adjusted for sex; age; MMSE score; BMI; education level; marital status; smoking status; alcohol consumption; energy intake; physical activity; employment status; depressive tendency; and history of hypertension, hyperlipidemia, ischemic heart disease, stroke, and diabetes. Fatty acid intake was divided into two groups (high, low) based on the median and according to sex. CLA was divided into two groups based on frequency of participation: “at least once or more per month” (high); and “once or several times per year or less” (low)

ARA, arachidonic acid; CLA, cognitive leisure activity; DHA, docosahexaenoic acid; EPA, eicosapentaenoic acid; LCPUFA, long-chain polyunsaturated fatty acids

### Odds ratios for cognitive decline according to the exposure combination

The exposure for the combination was art appreciation and each LCPUFA intake. The ORs of each combination were calculated using a category group as a reference, i.e., a low combination in the case of a low frequency of art appreciation and low fatty acid intake. In the crude model, the group with the combination of a high frequency of art appreciation and high intake of LCPUFA, DHA, EPA, and ARA had a significantly lower OR for cognitive decline. These significant associations were retained even in model I after adjusting for sex and age, and in model II after adjusting for all covariates (Table 5).

**Table 5:** Odds ratios (ORs) for cognitive decline according to the exposure combination

			Fatty acid			
Exposure combination			LCPUFA	DHA	EPA	ARA
			ORs (95% CIs)			
Crude Model						
Fatty acid intake (L)	x	Art appreciation (L)	1 (Reference)	1 (Reference)	1 (Reference)	1 (Reference)
Fatty acid intake (L)	x	Art appreciation (H)	1.03 (0.58–1.85)	0.98 (0.55–1.75)	0.93 (0.51–1.69)	1.01 (0.57–1.80)
Fatty acid intake (H)	x	Art appreciation (L)	1.01 (0.64–1.58)	1.09 (0.69–1.71)	1.06 (0.68–1.67)	0.96 (0.61–1.51)
Fatty acid intake (H)	x	Art appreciation (H)	0.29 (0.14–0.60)	0.32 (0.15–0.67)	0.37 (0.18–0.73)	0.26 (0.12–0.56)
Model I						
Fatty acid intake (L)	x	Art appreciation (L)	1 (Reference)	1 (Reference)	1 (Reference)	1 (Reference)
Fatty acid intake (L)	x	Art appreciation (H)	1.00 (0.55–1.82)	0.97 (0.54–1.75)	0.91 (0.50–1.67)	0.98 (0.54–1.76)
Fatty acid intake (H)	x	Art appreciation (L)	0.99 (0.62–1.57)	1.09 (0.69–1.74)	1.04 (0.66–1.66)	0.95 (0.60–1.51)
Fatty acid intake (H)	x	Art appreciation (H)	0.30 (0.15–0.63)	0.34 (0.16–0.71)	0.38 (0.19–0.76)	0.28 (0.13–0.60)
Model II						
Fatty acid intake (L)	x	Art appreciation (L)	1 (Reference)	1 (Reference)	1 (Reference)	1 (Reference)
Fatty acid intake (L)	x	Art appreciation (H)	0. 94 (0.47–1.86)	0.96 (0.48–1.90)	0.80 (0.40–1.60)	0.95 (0.49–1.85)
Fatty acid intake (H)	x	Art appreciation (L)	0.84 (0.49–1.42)	1.01 (0.59–1.71)	0.89 (0.53–1.51)	0.94 (0.55–1.60)
Fatty acid intake (H)	x	Art appreciation (H)	0.25 (0.11–0.56)	0.29 (0.13–0.65)	0.33 (0.15–0.72)	0.25 (0.10–0.59)

Note: Multiple logistic analysis

Model I: Adjusted for sex and age

Model II: Adjusted for sex and age; MMSE score; BMI; education level; marital status; smoking status; alcohol consumption; energy intake; physical activity; employment status; depressive tendency; and history of hypertension, hyperlipidemia, ischemic heart disease stroke and diabetes

Fatty acid intake was divided into two groups (high,low) based on the median values according to sex. Art appreciation was divided into two groups based on the frequency of participation: “at least once or more per month” (high); “once or several times per year or less” (low)

ARA, arachidonic acid; CI, confidence interval; DHA, docosahexaenoic acid; EPA, eicosapentaenoic acid; L, low; LCPUFAs, long-chain polyunsaturated fatty acids; H, high; OR, odds ratio

## Discussion

To the best of our knowledge, this is the first study to demonstrate the potential impact of the interaction of varied and high-frequency engagement in CLAs with high LCPUFA intake on the preservation of cognitive function in community-dwelling older individuals. One of the CLAs, art appreciation, showed a significant interaction in combination with LCPUFA intake. In the main effect model, both art appreciation and LCPUFA intake showed significant associations with cognitive decline, respectively. The combination of a high frequency of art appreciation and high LCPUFA intake yielded a lower OR for cognitive decline compared with a low combination of the two parameters. DHA, EPA, and ARA exhibited the same tendency.

Several clinical research articles have shown that CLAs are more effective for the preservation of cognitive function than physical or social activities [28, 35]. According to animal studies, the learning inability and brain β-amyloid deposition were suppressed in cases involving complex play equipment in addition to exercise compared with the exercise-only group [36]. Cognitive function gradually declines in older people, who have fewer opportunities to use their brains [37]. Continued engagement in CLAs in later life may stabilize or activate cognitive function [38]. Previous studies have shown that frequent participation in various activities that provide cognitive stimulation was associated with a reduced risk for Alzheimer’s disease [39]. In line with this, the present study found that participation in six CLAs had a significant main effect on cognitive decline. As such, CLAs alone may prevent cognitive decline, provided that patients frequently participate in various CLAs.

Art appreciation creates a dialog with others and invites the exchange of impressions; thus; art appreciation could be considered a social activity as well as an intellectual activity. Since the relationship between social connections and cognitive function and Alzheimer’s disease pathology has been reported, the social activity component of art appreciation may also be reflected in our results [40, 41]. Art appreciation could also positively influence emotions and mood. Overall, it was observed that those who engaged in five or more CLAs and those who frequently engaged in art appreciation tended to have healthier lifestyles than those with less frequent participation in these activities. One recent study reported longitudinal associations between receptive arts engagement and mortality [42]. Another study suggested that positive emotions help control cardiovascular systems linked with dementia risk [43]. An interventional study showed that exercise combined with music compared with exercise alone preserved cognitive function in older people [44]. Therefore, it is inferred that the positive emotions derived from art appreciation contribute to cognitive function.

The brain’s emotional areas, such as the amygdala and the entire orbital insular cortex, are activated during art appreciation [45, 46]. According to a study, the same areas of the brain were reportedly active during both visual and auditory means of art appreciation [47]. Another study highlighted the importance of hippocampal−orbital insular cortex interactions [48]. Brain regions that are connected to emotions or memory function, such as the orbitofrontal cortex or hippocampus, respectively, showed lower LCPUFA levels with aging [8–10]. LCPUFAs are well-known cannabinoid precursors. The function of the brain’s endogenous cannabinoid system diminishes with normal aging [49–52]. According to a recent report, the administration of low-dose cannabinoids to young mice impairs the ability to perform learning and memory tasks; however, the same dose can improve the ability to perform learning and memory tasks in aged mice [53]. Therefore, the reduction of LCPUFA levels that accompanies aging may influence the cognitive function of the part of the brain that is responsible for emotions and memory. Art appreciation may activate emotional parts of the brain that are linked to the memory parts.

A significant interaction between art appreciation and EPA was not observed in the present study. EPA certainly is less abundant in the brain than are DHA and ARA; however, a meta-analysis demonstrated that EPA may be more effective than DHA in the treatment of mood disorders, such as depression [54]. Each LCPUFA might provide an original contribution to the preservation of cognitive function.

The combination of CLA engagement and LCPUFA intake is more effective in preventing cognitive decline than either intervention alone. These two interventions may support the cognitive reserve in different ways, mimicking the functions of software and hardware [55]. LCPUFA intake supplements brain components that decline with age, contributing to the maintenance of structural stability (hardware), whereas CLAs strengthen neural circuit functions (software); thus, the functions of the hardware can be considered fully utilized because of the existence of software. As such, CLAs that can continue to functionally activate various areas of the brain would be a useful leisure activity for the brain.

### Strengths and limitations

The study participants were randomly selected among community dwellers based on resident registrations. Accurate nutritional assessments were based on 3-day dietary intake and were recorded using a scale and photographs. However, the limitations of this study should be considered. First, our findings may not be applicable to other countries or cultures because the present results were obtained using the Japanese cohort population. Japanese people consume plenty of fish and shellfish, which are the primary sources of EPA and DHA [56, 57]. The EPA and DHA intake levels might contribute more to the LCPUFA intake level because ARA intake is similar among different countries [57]. Japanese people may have a higher LCPUFA intake than other populations. Moreover, CLAs vary widely between countries due to cultural differences. However, art appreciation may be a universal activity if the esthetic sense is a commonality among different cultures.

Second, it is unclear whether the delimitations and category of the CLAs used in this study are typical or reasonable. The delimitation and frequency of activities differ from one report to another. In the present study, the interaction between LCPUFA and art appreciation was significant in various delimitations. However, since the validity of the activity delimitations has not been established, we cannot refer to a specific activity frequency. Another serious limitation is that the frequency of activities was uniform across all CLAs in this analysis. For example, reading books once a month or more is not considered as a high frequency. Dividing the frequency of activities by the median number of people is one method for addressing this challenge; however, some items, such as newspapers, cannot be divided evenly. Furthermore, there is substantial variation between reports regarding separations for different types of activities. Therefore, we conducted our analysis according to the delimitations described in a report that analyzed items of intellectual activities similar to those assessed in our study by uniformly dividing the frequency of activities into two parts [34]. As for the classification of CLAs, we developed our questionnaire with reference to a study that surveyed leisure activities in older adults [27] and various administrative surveys. In this questionnaire, for example, calligraphy, painting, photography, and Japanese dressmaking are all included within the category of creative activities. Therefore, the relationship between individual activities within that category and the maintenance of cognitive function is not clear.

Third, the MMSE is a method of evaluation that is used to screen for dementia and is not a diagnostic tool for MCI and cognitive decline. At present, various cut-off values are used for provisionally determining MCI and cognitive decline. The difference between the clinical diagnosis and the definition based on MMSE cut-off score should be considered.

Fourth, although our study was longitudinal, the possibility of causal inversion cannot be ruled out completely. Because dementia and depression are reportedly associated with each other, participation in leisure activities might be reduced in people in the early stages of cognitive impairment [58, 59]. The decrease in the frequency of a CLA might reflect the early symptoms of dementia.

Fifth, although a significant interaction was observed between LCPUFA intake and engagement in ≥ 5 CLAs, no interaction was noted between LCPUFA intake and participation in each of the four CLAs other than art appreciation. The CLAs other than art appreciation included reading newspapers, reading books, writing, and doing yard work. The participants in the present study participated in these four CLAs relatively frequently given that the aforementioned activities are familiar to older people in Japan [60–62]. Therefore, the significant association observed between five or more CLAs and cognitive function observed in the present study may be attributed to the addition of art appreciation to the four familiar and frequently performed intellectual activities.

Sixth, the validity of the nutritional assessment for quantifying LCPUFA intake is insufficient. Fukumoto et al. [63] reported that a seven-day dietary record is required to estimate the true value of PUFA, even within an error range of ± 15 %. We used a three-day dietary record, which may not have provided an accurate estimation of LCPUFA intake. For example, the total value of DHA and EPA was 0.963 g in our study, but Kawabata et al. [64] reported 0.90 g for men and 0.75 g for women in a 28-day dietary record survey of older adults in Japan; thus, the possibility of overestimation due to using a three-day dietary record cannot be denied. However, for ARA, the mean value for both men and women in this study was 0.161 g, and the 28-day dietary survey reported similar values of 0.15 g for women and 0.17 g for men.

### Implications for practice

Similar to muscles, based on the use-it-or-lose-it theory, cognitive function can undergo a gradual decline in older people, who have increasingly fewer opportunities to use these skills. Ongoing participation in CLAs later in life might stabilize or even activate the unused aspects of cognitive functioning. A recent prospective study revealed an association between limited participation in cognitive or social activities and an increased risk of developing dementia [65]. Daily participation in CLAs may also have a positive impact on mood and emotions, in addition to preserving cognitive function. Moreover, many opportunities are available to modify the daily diet regardless of age because the diet is essential for life. It is possible that older people in Japan experience a higher intake of LCPUFAs because of the substantial role played by fish in the indigenous diet. Older people in other countries might be encouraged to include more fish in their daily diets.

## Conclusions

The present study demonstrated the interaction of the high-frequency engagement in a variety of CLAs combined with high LCPUFA intake on cognitive function preservation in community-dwelling older people. In addition, one of the CLAs, i.e., art appreciation, may be associated with the preservation of cognitive function. These lifestyle factors can be modified, and CLAs in particular are better suited for older people because of their lower physical burden. Further study is required to establish a link with clinical significance, which may also provide modifiable daily interventions to maintain cognitive function.

## Data Availability

The datasets generated and analyses performed as part of the current study are not publicly available due to the consent requirements of the participants. However, participants characteristics, including sex and age-stratified descriptive data, are available from the corresponding author upon reasonable request.

## Abbreviations

ARA: Arachidonic acid
BMI: Body mass index
CI: Confidence intervals
CLA: Cognitive leisure activities
DHA: Docosahexaenoic acid
EPA: Eicosapentaenoic acid
LCPUFA: Long-chain polyunsaturated fatty acids
MCI: Mild Cognitive Impairment
MET: Metabolic equivalents
MMSE: Mini-Mental State Examination
MUFA: Monosaturated fatty acids
NILS-LSA: National Institute for Longevity Sciences-Longitudinal Study of Aging
OR: Odds ratio
SFA: Saturated fatty acids
